# Soluble uric acid induces myocardial damage through activating the NLRP3 inflammasome

**DOI:** 10.1111/jcmm.15523

**Published:** 2020-06-18

**Authors:** Hailong Zhang, Yuting Ma, Run Cao, Guanli Wang, Shaowei Li, Yue Cao, Hao Zhang, Meichen Liu, Guangchao Liu, Jun Zhang, Shulian Li, Yaohui Wang, Yuanfang Ma

**Affiliations:** ^1^ Joint National Laboratory for Antibody Drug Engineering Key Laboratory of Cellular and Molecular Immunology of Henan Province School of Basic Medicine Henan University Kaifeng China; ^2^ Clinical Laboratory Huaihe Hospital Henan University Kaifeng China

**Keywords:** myocardial damage, NLRP3 inflammasome, soluble uric acid, TLR6/NF‐κB signal pathway, UCP2

## Abstract

Uric acid crystal is known to activate the NLRP3 inflammasome and to cause tissue damages, which can result in many diseases, such as gout, chronic renal injury and myocardial damage. Meanwhile, soluble uric acid (sUA), before forming crystals, is also related to these diseases. This study was carried out to investigate whether sUA could also activate NLRP3 inflammasome in cardiomyocytes and to analyse the mechanisms. The cardiomyocyte activity was monitored, along with the levels of mature IL‐1β and caspase‐1 from H9c2 cells following sUA stimulus. We found that sUA was able to activate NLRP3 inflammasome, which was responsible for H9c2 cell apoptosis induced by sUA. By elevating TLR6 levels and then activating NF‐κB/p65 signal pathway, sUA promoted NLRP3, pro‐caspase 1 and pro‐IL‐1β production and provided the first signal of NLRP3 inflammasome activation. Meanwhile, ROS production regulated by UCP2 levels also contributed to NLRP3 inflammasome assembly and subsequent caspase 1 activation and mature IL‐1β secretion. In addition, the *tlr6* knockdown rats suffering from hyperuricemia showed the lower level of IL‐1β and an ameliorative cardiac function. These findings suggest that sUA activates NLRP3 inflammasome in cardiomyocytes and they may provide one therapeutic strategy for myocardial damage induced by sUA.

## 1 INTRODUCTION

NOD‐like receptor (NLR) family pyrin domain containing 3 (NLRP3) inflammasome is a multiprotein complex, containing NLRP3, apoptosis‐associated speck‐like protein containing a CARD (ASC) and caspase‐1.^1^, ^2^, ^3^ When the host receives inflammatory stimuli, the levels of NLRP3, pro‐IL‐1β and pro‐caspase 1 will be elevated through NF‐κB or MAPK signal pathways, which provide the first signal of NLRP3 inflammasome activation.^4^, ^5^, ^6^ Then, PAMPs and DAMPs, such as uric acid (UA) crystal, ATP, ROS and cholesterol, will provide the second signal, which produce NLRP3 inflammasome.^7^, ^8^, ^9^, ^10^, ^11^ NLRP3 inflammasome promotes pro‐caspase 1 maturation and then proceeds to cleave pro‐IL‐1β and pro‐IL‐18 into mature IL‐1β and IL‐18.^1^, ^4^

NLRP3 inflammasome activation not only plays an important role in anti‐infection, but also involves in many autoimmunity diseases, diabetic cardiomyopathy, Alzheimer and metabolic diseases.^12^ Hyperuricemia is one kind of common metabolic diseases, which is characterized by high levels of uric acid in serum, and the accumulation of UA crystal in the joints, kidneys and hearts.^13^ UA crystal is a kind of DAMPs, which can abnormally activate the immune system and then induces tissue damage and diseases, such as gout, chronic renal injury and cardiovascular diseases.^14^, ^15^, ^16^, ^17^ Through activating NF‐κB and MAPK signal pathways, UA crystal induces the release of proinflammatory cytokines, such as IL‐1β and IL‐18, which are mainly regulated by NLRP3 inflammasome activation.^6^, ^15^, ^18^ Moreover, through phagocytosis without the involvement of the cellular receptor, UA crystal can induce lysosomal damage, and then activates NLRP3 inflammasome.^19^, ^20^

Recently, some researches have shown that soluble serum UA levels are also related to inflammatory reaction and tissue damage, which result in diseases such as chronic renal disease and gout.^19^, ^21^, ^22^ A high level of sUA in the normal range can induce renal function loss in patients with diabetes.^23^, ^24^ Moreover, sUA primes proinflammatory cytokine production through TLRs in gout‐related disease. The mechanisms may be associated with the activation of NF‐κB and inhibition of IL‐1Rα.^25^, ^26^ However, it is still unknown whether sUA activates NLRP3 inflammasome and takes part in cardiocyte damage.

In this study, we investigated whether sUA could activate NLRP3 inflammasome in H9c2 cells and analysed the mechanisms of NLRP3 inflammasome activation. Our study identified that sUA was able to activate NLRP3 inflammasome and then induced cardiomyocyte apoptosis. We further observed that through TLR6/NF‐κB/p65 signal pathway, sUA provided the first signal of NLRP3 inflammasome activation. Meanwhile, sUA induced ROS production depending on decreasing UCP2 levels, which provided the second signal of NLRP3 inflammasome activation. Furthermore, unlike UA crystal, sUA activated NLRP3 inflammasome independent of lysosome damage. In the end, we took advantage of *tlr6* knockdown rats suffering from hyperuricemia and observed that *tlr6* knockdown improved myocardial damage and left ventricular remodelling induced by sUA.

## 2 MATERIALS AND METHODS

### 2.1 Rats

Wistar rats (6‐8 weeks old) were obtained from Beijing Vital River Laboratory Animal Technology Co. Ltd and fed in sterile animal houses. All animal experiments were authorized by the Animal Experimental Ethics Committee of Henan University. The animal experiments in vivo were described in Methods S1.

### 2.2 Cell culture and treatment

H9c2 cells were obtained from the Library of Typical Culture of the Chinese Academy of Sciences (Shanghai, China), which were maintained with the DMEM medium containing 5.5 mM glucose, 10% FBS (V/V), 100 U/mL penicillin and 100 g/L streptomycin. In addition, the cells were treated as described below.

### 2.3 Cell vitality and apoptosis

H9c2 cells were stimulated with different concentrations of UA (UA, 50, 100, 200 and 400 mg/L; Sigma). Several hours later (12, 24 and 48 hours), one cytotoxicity detection kit (LDH; Merck) was used to detect cellular damage with the supernatant. Meanwhile, another cell proliferation and cytotoxicity assay kit (MTS) was used to detect cell vitality according to the instruction. The cell apoptosis was assayed with Annexin V‐FITC/PI apoptosis detection kit, which was described in previous publications.^12^ For caspase 1 inhibitor assay, Z‐YVAD‐FMK (YVAD) was added into the supernatant of H9c2 cells along with 200 mg/L UA. Twenty‐four hours later, LDH and MTS were used to detect cell vitality. In addition, after treated with 200 mg/L UA for 24 hours, cell vitality of H9c2 cells NLRP3 knockdown was detected with LDH and MTS.

### 2.4 Western blot

Briefly, the proteins from H9c2 cells or heart tissues were separated by 12% SDS‐polyacrylamide gels and transferred onto PVDF membranes. After blocked with TBST containing 5% BSA, membranes were incubated with NLRP3, ASC, TLR6 (Santa Cruz), Pro‐IL‐1β, Pro‐Caspase 1 (abcam), mIL‐1β, Cleaved Caspase‐1, p65, p‐p65, IKKα, IKKβ, p‐IKKα/β, p‐TAK1, TAK1, p‐JNK, JNK, p‐MKK3/6, MKK6, p‐p38, p38, VDAC (Cell Signaling Technology Inc), Cytochrome C (Bioword), UCP2 (Proteintech) rabbit antibodies and GAPDH (ABclonal Technology) mouse antibody overnight at 4℃. Then, the membranes were incubated with horseradish peroxidase‐labelled secondary antibodies (ABclonal Technology) for 2 hours at room temperature. Subsequently, the protein bands were detected with Pierce™ ECL Western Blotting Substrate and scanned by an automatic chemiluminescence imaging system (Tanon 5200; Tanon).

### 2.5 Real‐time fluorescence quantification PCR

After total RNA was obtained from H9c2 cells and heart tissues, real‐time fluorescence quantification PCR (RT‐qPCR) was performed for detecting the levels of *Gapdh*, *Nlrp3, Caspase 1, Il1b, Tlr1, Tlr2, Tlr3, Tlr4, Tlr5, Tlr6, Tlr7, Tlr8*, *Tlr9*, *Tspo, Slc25a1* and *VDAC* genes. PrimeScript™ RT Master Mix (Takara) was used to synthesize cDNA (Table S1), and RT‐qPCR was finished with SYBR™ Select Master Mix (Thermo Fisher). The levels of target genes were automatically normalized the level of *Gapdh*. The data were presented as relative fold change with respect to the control sample.

### 2.6 Knockdown and overexpression

For knockdown with siRNA, 1 × 10^5^ cells were cultured in cell cultured plate with 6 holes. Six hours later, the cells were transfected with siRNA targeted *Nlrp3* or *Tlr6* (RiboBio Co.), according to the manufacturer's instructions. Twenty‐four hours later, the expression of NLRP3 or TLR6 was detected by western blot or Immunofluorescence.

For overexpression, 1 × 10^5^ cells were cultured in cell cultured plate with six holes. Twelve hours later, the supernatant was discarded and the cells were transfected with lentivirus containing UCP2 (LV‐UCP2, MOI = 20). Twenty‐four hours later, the supernatant was discarded and the cells were sequentially cultured with fresh medium for 24 hours. At last, the expression of UCP2 was detected by western blot.

### 2.7 Immunofluorescence

Immunofluorescence was used to detect the level of TLR6, UCP2 and p65 proteins.^12^ Briefly, 1 × 10^5^ cells were cultured in Glass Bottom Bell Culture Dish (20 mm polystyrene Non‐pyrogenic Sterile). After treated with several drugs, the supernatants of H9c2 cells were discarded and the cells were washed with PBS for three times. Then, the cells were fixated and permeabilized with 4% paraformaldehyde containing 0.2% Triton X‐100 for 20 min. After washed with PBS for three times, the cells were blocked with 5% BSA for 1 hour at 37℃ and subsequently incubated with TLR6 rabbit antibody overnight at 4℃. After washed with PBS for three times, the cells were incubated with Alexa Fluor 488 conjugated anti‐rabbit IgG antibody for 2 hours. Then, the cells were incubated with DAPI for 5 min. At last, the cells were washed with PBS and photographs taken by fluorescence inverted microscope for TLR6 and UCP2 (NIKON). Meanwhile, laser scanning confocal microscope (Zeiss) was used to detect p65.

### 2.8 Reactive oxygen species

The peroxide‐sensitive fluorescent probe 2′, 7′‐dichlorofluorescein diacetate (DCFH‐DA) was used to detect the levels of reactive oxygen species (ROS). H9c2 cells were treated with different concentrations of UA, or 200 mg/L UA along with several concentrations of NAC for 24 hours. Then, the cells were harvested and washed with PBS. After incubated with DCFH‐DA for 30 min, the cells were detected by flow cytometer to analyse the levels of ROS. Meanwhile, the cells transfected with LV‐UCP2 or null control were treated with 200 mg/L UA for 24 hours. The levels of ROS were also detected as mentioned above.

### 2.9 Lysosomal damage

H9c2 cells were stimulated with 200 mg/L UA for 24 hours, and then incubated with LysoTracker Green DND‐26 (Thermo Fisher Scientific) or Alexa Fluor 647 Conjugated dextran (Thermo Fisher Scientific) for an additional 2 hours. After washed with PBS, H9c2 cells were incubated with hoechst for 5 min. Then, laser scanning confocal microscope (Zeiss) was used to detect lysosomal damage.

### 2.10 Statistical analysis

All values were shown as mean ± SEM of three independent experiments. Statistical significance among two groups was performed with two‐tailed Student's *t* test. For three and more groups, one‐way ANOVA was used on GraphPad Prism 6.0 software, followed by post hoc testing (Tukey test). *P* ≤ 0.05 was considered as significant.

## 3 RESULTS

### 3.1 sUA induced cell apoptosis in cardiomyocytes and that mainly relied on NLRP3 inflammasome activation

Firstly, we incubated H9c2 cells in presence of several concentrations of sUA for 12, 24 or 48 hours. Although sUA could induce H9c2 cells to release more LDH than normal group (0 mg/L UA), there were no differences among these groups within a short period of time (12 hours) (Figure S1A). Meanwhile, MTS assay also showed that sUA could not induce H9C2 cell damage (Figure S1B). While, flow cytometry (FACS) showed that sUA induced H9C2 cell apoptosis, which was dose‐dependent (Figure S1C, D). After H9C2 cells were treated for 24 or 48 hours, we observed that sUA dramatically induced H9C2 cell damage and cell apoptosis in the dose‐dependent and time‐dependent manners (Figure S1A‐D).

**FIGURE 1:**
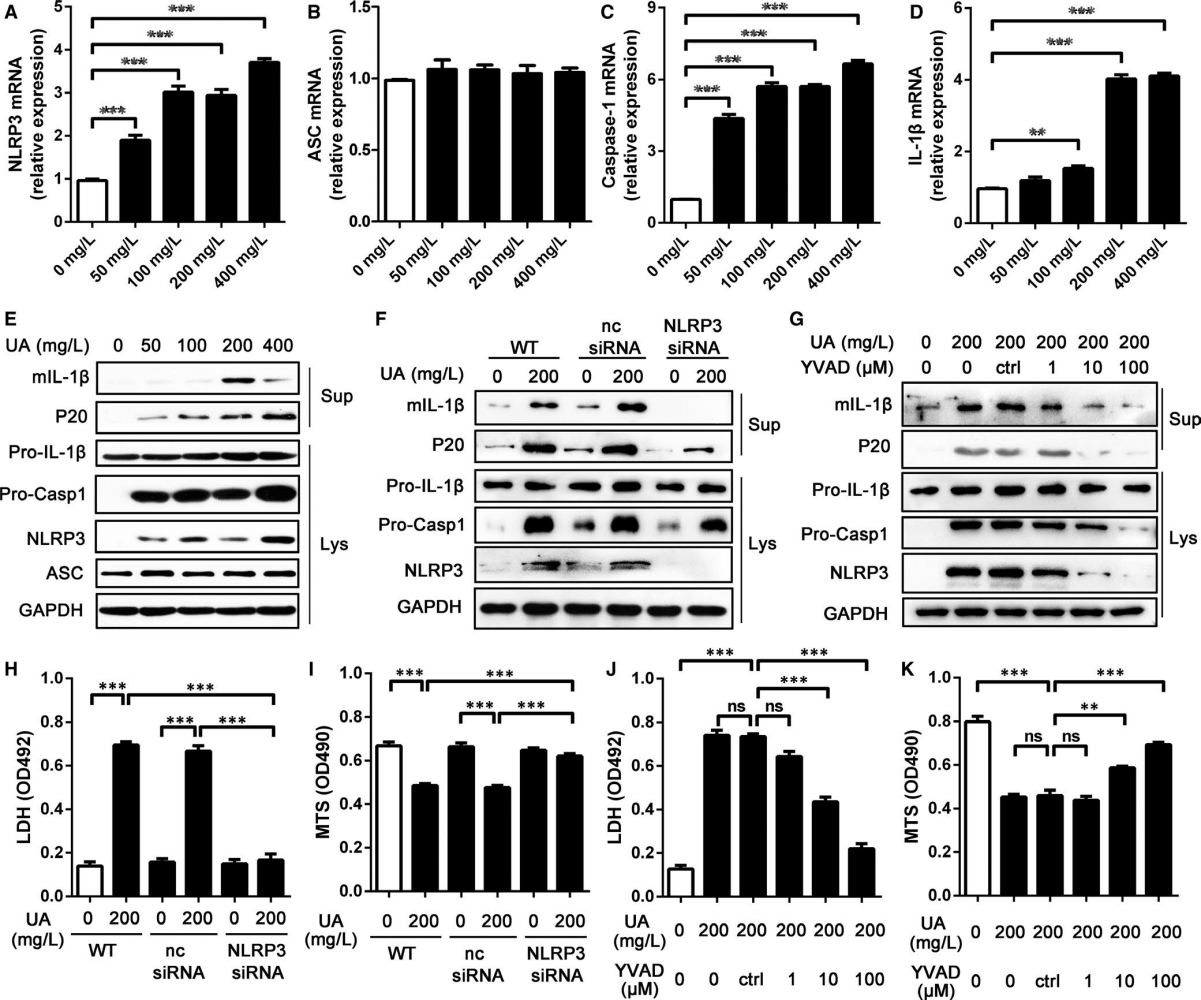
H9c2 cell damage induced by sUA was mainly relied on NLRP3 inflammasome activation. A‐D, The gene levels of NLRP3 inflammasome components. After H9c2 cells were stimulated with sUA for 24 h, the levels of *NLRP3* (A), *Asc* (B), *Caspase 1* (C) and *Il1b* (D) mRNA were detected by RT‐qPCR and normalized to GAPDH. E, The medium supernatants (Sup) and cell lysates (Lys) of H9c2 cells treated with serial sUA were analysed by WB. F, Silencing NLRP3 inhibited NLRP3 inflammasome activation in H9c2 cells. G‐H, LDH (G) and MTS (H) were executed to detect H9c2 cell damage after that NLRP3 was knocked down. I, The protein levels of NLRP3 inflammasome components were detected in H9c2 cells treated with sUA and YVAD for 24 h. J‐K, LDH (J) and MTS (K) were executed to detect cell damage in H9c2 cells treated with sUA and YVAD for 24 h. Data are shown as mean ± SEM. Ns means no statistical differences, **P ≤ 0.01, ***P ≤ 0.001

To determine the mechanisms of cardiocyte damage induced by sUA, the activation of NLRP3 inflammasome was firstly detected. We incubated H9c2 cells with sUA for 24 hours. We observed that sUA significantly induced the expression of *NLRP3, Caspase 1* and *Il1b* mRNA compared with non‐stimulated cells, and this effect was dose‐dependent, while *Asc* had no change (Figure 1A‐D). Meanwhile, the protein levels of NLRP3 and pro‐IL‐1β in H9C2 cells treated with sUA were higher than that in non‐stimulated cells (Figure 1E, F). Matured IL‐1β (mIL‐1β) levels were enhanced in the supernatant after sUA stimulation for 24 hours (Figure 1E, F). We also observed that inflammasome activation by sUA was NLRP3 dependent, as H9c2 cells transfected with NLRP3‐siRNA secreted lower mIL‐1β compared to that transfected with NC‐siRNA (Figure 1F). Moreover, when the NLRP3 levels in H9C2 cells were knocked down, the cardiocyte damage induced by sUA was significantly improved, which was measured by MTS and LDH assay (Figure 1G, H).

Inflammasome activation by sUA was simultaneously confirmed by the detection of caspase‐1. First, sUA stimulated P20 production in a dose‐dependent manner (Figure 1E). Meanwhile, P20 levels were reduced in cells transfected with NLRP3‐siRNA compared to the controls (Figure 1F). Second, when H9c2 cells were treated with serious concentrations of YVAD in the presence of sUA, mIL‐1β and P20 levels in supernatant were significantly reduced, which was dose‐dependent (Figure 1I). Moreover, NLRP3 and pro‐caspase 1 levels treated with sUA and YVAD were lower than that treated with sUA alone (Figure 1I). Last, the cardiocyte damage induced by sUA was also reduced by YVAD (Figure 1J, K). Therefore, these data indicated that sUA‐induced NLRP3 inflammasome activation was answerable for H9c2 cell damage.

### 3.2 sUA induced NLRP3 inflammasome activation through TLR6/NF‐κB/p65 signal pathway

**FIGURE 2:**
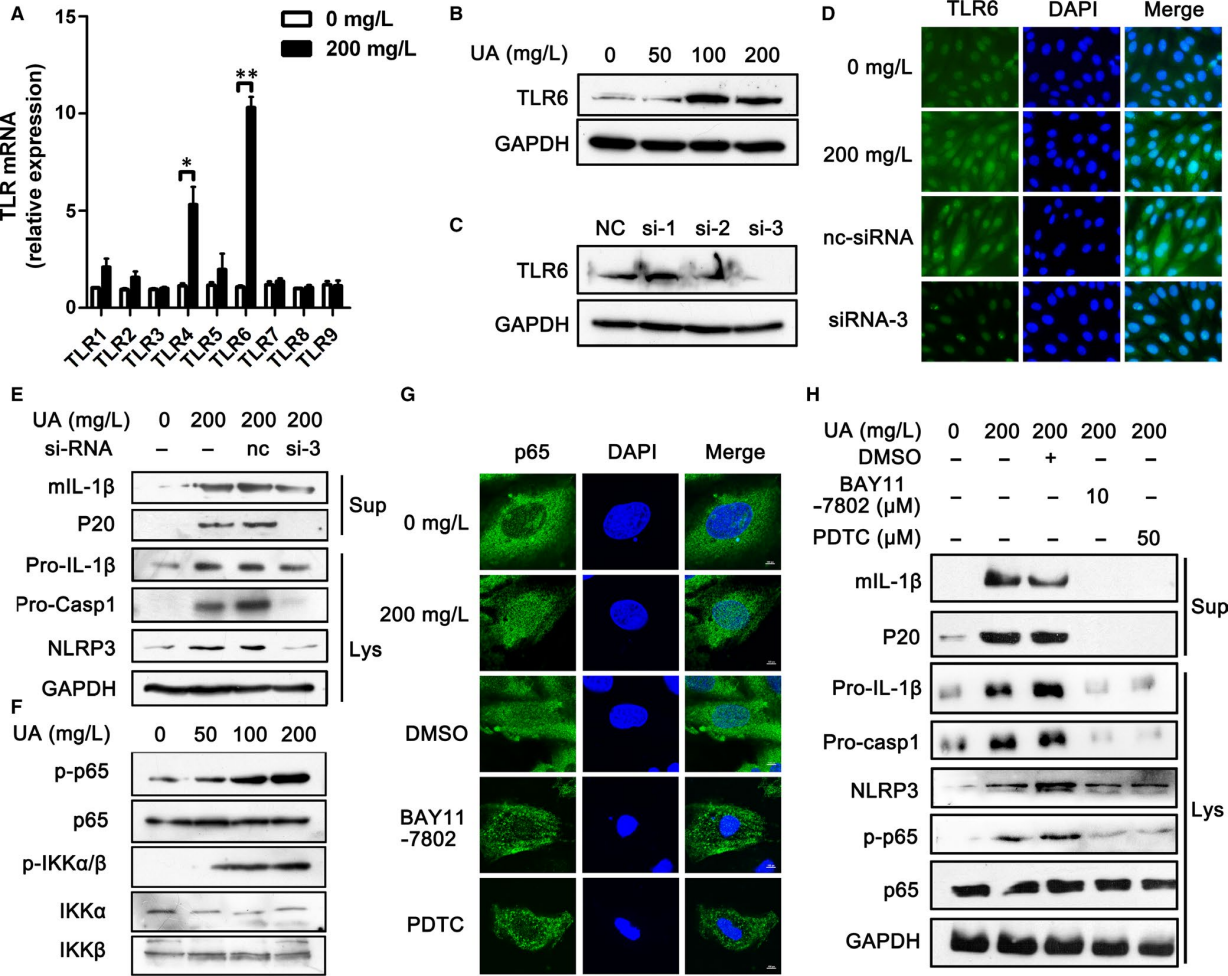
NLRP3 inflammasome activation induced by sUA was mainly depended on TLR6/ NF‐κB/p65 signal pathway. A, The gene levels of toll‐like receptors were detected by RT‐qPCR after that H9c2 cells were treated with sUA for 24 h. B, The protein level of TLR6 was analysed by WB after that H9c2 cells were treated with sUA for 24 h. C, The protein level of TLR6 was analysed by WB after that H9c2 cells were silenced by TLR6‐targeted siRNA for 24 h. D, The protein level of TLR6 was analysed by IF after that H9c2 cells silenced by TLR6‐targeted siRNA were stimulated with sUA. E, The activation of NLRP3 inflammasome was analysed by WB in H9c2 cells silenced by TLR6‐targeted siRNA. F, The protein levels of p‐p65, p65, p‐IKKα/β, IKKα and IKKβ were analysed by WB in H9c2 cells treated with sUA. G, The location of p65 in H9c2 cells treated with sUA and NF‐κB inhibitors. H, The activation of NLRP3 inflammasome in H9c2 cells treated with sUA and NF‐κB inhibitors. Data are shown as mean ± SEM. *P ≤ 0.05, **P ≤ 0.01

The activation of innate immunity, which induces the generation of NLRP3, pro‐IL‐1β and pro‐caspase 1 proteins, is the necessary prerequisite for NLRP3 inflammasome activation. In Figure 1, we found that sUA could up‐regulate the gene and protein levels of NLRP3, pro‐IL‐1β and pro‐caspase 1, which implied that sUA could activate the innate immunity. To ascertain the mechanisms by which sUA activated the innate immunity, the gene levels of toll‐like receptors were detected by RT‐qPCR after that H9c2 cells were treated with 200 mg/L UA for 24 hours. We found that sUA significantly elevated the levels of *tlr4* and *tlr6* gene, while the levels of *tlr1*, *tlr2*, *tlr3*, *tlr5*, *tlr7*, *tlr8* and *tlr9* gene had no changes (Figure 2A). The role of TLR4 in NLRP3 inflammasome activation stimulated by sUA has been expounded in several publications,^22^, ^27^ while the role of TLR6 in NLRP3 inflammasome activation remains poorly understood. In an attempt to address the role of TLR6 in NLRP3 inflammasome activation stimulated by sUA, we studied the protein level of TLR6 by WB. sUA could increase TLR6 expression in H9c2 cells after a 24 hours stimulation with several concentrations of UA, which was dose‐dependent (Figure 2B). Moreover, when the level of TLR6 was knockdown in H9c2 cells with siRNA, the protein levels of NLRP3, pro‐IL‐1β and pro‐caspase 1 were lower compared with the control ones (Figure 2C‐E). In addition, contrary to sUA treated H9c2 cells, TLR6 knockdown cells displayed a decrease in the levels of mIL‐1β and P20 (Figure 2E). When the TLR6 levels in H9c2 cells were knocked down, the cardiocyte damage induced by sUA was significantly improved, which was measured by LDH and MTS assay (Figure S2A, B). Meanwhile, we also found decreased apoptosis rate in H9c2 cells transfected with siRNA when compared with NC (Figure S2C, D). These data indicated that NLRP3 inflammasome activation induced by sUA was mainly dependent on TLR6.

To ultimately identify the mechanism of NLRP3 inflammasome activation induced by sUA, the downstream signal pathways were analysed. We found that sUA did not affect the phosphorylation levels of TAK and JNK, and the total levels of TAK and JNK had no change (Figure S3A‐C). Similarly, we observed that the levels of p‐MKK3/6, p‐p38, MKK6 and p38 in H9c2 cells treated with sUA had no change compared to untreated cells (Figure S3D‐F). These data indicated that NLRP3 inflammasome activation induced by sUA was independent of TAK1/MKK4/JNK and MKK6/p38 MAPK signal pathways. Then, we confirmed that NLRP3 inflammasome activation was because of the NF‐κB/IKK/p65 signal pathway, as sUA up‐regulated the levels of p‐p65 and p‐IKKα/β (Figure 2F). In addition, sUA promoted p65 into the cell nucleus, which was decreased by NF‐κB inhibitors (BAY11‐7802 and PDTC) (Figure 2G). Besides these, BAY11‐7802 and PDTC decreased the levels of p‐p65, NLRP3, pro‐IL‐1β and pro‐caspase 1 (Figure 2H). The levels of mIL‐1β and P20 in the supernatant were lower in H9c2 cells treated with NF‐κB inhibitors than that treated with DMSO (Figure 2H).

### 3.3 sUA induced NLRP3 inflammasome activation in a mitochondrial ROS‐dependent manner

**FIGURE 3:**
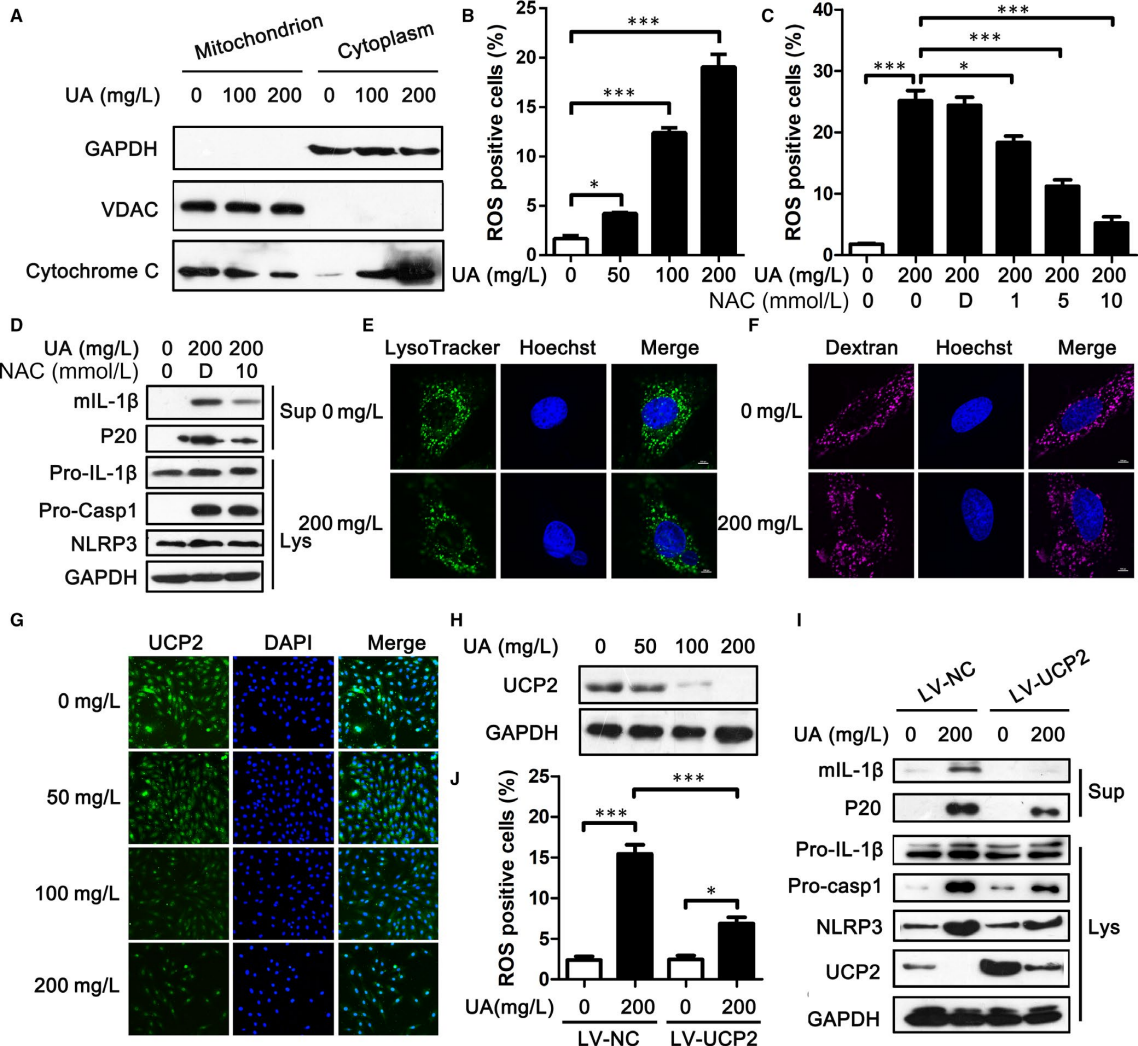
Soluble uric acid (sUA) induced NLRP3 inflammasome activation in a mitochondrial ROS‐dependent manner regulated by UCP2. A, The cytochrome *c* in the cytoplasm and mitochondrion of H9c2 cells was detected by WB. B, ROS level was detected by FCM in H9c2 cells treated with sUA for 24 h and the statistical result of positive cells. C, ROS level was detected by FCM in H9c2 cells treated with sUA and NAC for 24 h and the statistical result of positive cells. D, The activation of NLRP3 inflammasome was detected in H9c2 cells treated with sUA and NAC for 24 h. E‐F, Confocal microscopy of H9c2 cells stimulated for 24 h with sUA and then incubated with LysoTracker Green DND‐26 (E) or Alexa Fluor 647 conjugated dextran (F) for 2 h. Cell nuclei were stained with Hoechst dye (blue). G‐H, The protein level of UCP2 was analysed by IF (G) and WB (H) in H9c2 cells treated with sUA. I, The activation of NLRP3 inflammasome was detected in H9c2 cells transfected with LV‐UCP2. J, ROS level was detected by FCM in H9c2 cells transfected with LV‐UCP2 and the statistical result of positive cells. Data are shown as mean ± SEM. *P ≤ 0.05, ***P ≤ 0.001

In an attempt to address the second signal of NLRP3 inflammasome activation induced by sUA, we analysed the mitochondrial damage through detecting the cytochrome *c* release into the cytoplasm. sUA could induce cytochrome *c* release into the cytoplasm, which was dose‐dependent (Figure 3A). In addition, we analysed ROS production by staining H9c2 cells with the fluorescent probe DCFH‐DA. DCFH‐DA was hydrolysed to produce DCFH in cells, and oxidation of DCFH produced green fluorescence. Contrary to WT cells, H9c2 cells treated with sUA displayed an increase in green fluorescence, which was dose‐dependent (Figure 3B).

When the H9c2 cells were stimulated with different concentrations of NAC (a reductant inhibits ROS production), the DCFH fluorescence induced by sUA was decreased (Figure 3C). Meanwhile, NAC decreased the protein levels of mIL‐1β and p20 in the supernatant (Figure 3D). However, the protein levels of pro‐IL‐1β, pro‐caspase 1, NLRP3 and ASC had no change in H9c2 cells treated with NAC and sUA compared to sUA alone (Figure 3D).

UA crystals can activate NLRP3 inflammasome through lysosomal damage. To detect whether sUA could induce lysosomal damage, we used confocal reflection microscopy to lysosomal integrity. LysoTracker Green DND‐26 is a green fluorescent dye, which can stain acidic compartments, such as lysosome. When the lysosome is damaged, no green fluorescence is detected. In untreated H9c2 cells, we found lots of green fluorescence spots in the cytoplasm, as expected. Meanwhile, there were still many green fluorescence spots in most cells exposed to sUA (Figure 3E). In addition, Alexa Fluor 647 Conjugated dextran, which was ingested and transported through the lysosomal pathway, also stained normal lysosomal compartments and showed lysosomal location in most cells (Figure 3F). These results indicated that sUA activated NLRP3 inflammasome independent of lysosomal damage, which was related to the generation of mitochondrial ROS.

### 3.4 ROS production induced sUA was mainly dependent on UCP2

In order to detect the mechanisms of ROS production induced by sUA, we analysed UCP2 protein level in H9c2 cells treated with sUA. UCP2 is a mitochondrial protein, which has been identified to prevent ROS generation. By immunofluorescence and immunoblotting, we found that sUA significantly inhibited UCP2 production, which was dose‐dependent (Figure 3G, H). Then, H9c2 cells were transfected with LV‐UCP2 for 24 hours. We observed an up‐regulation of UCP2 protein levels in H9c2 cells transfected with LV‐UCP2 (Figure 3I). When the levels of UCP2 protein were up‐regulated, ROS production induced by sUA was significantly inhibited (Figure 3J). Besides UCP2, some mitochondrial proteins, such as the translocator protein (TSPO), tricarboxylate transport protein (Slc25a1) and VDAC, can also regulate ROS generation. However, we found that the gene levels of TSPO, Slc25a1 and VDAC had no obviously changes in H9c2 cells treated with sUA (Figure S4A‐C). Meanwhile, sUA did not change the protein level of VDAC (Figure 3A).

Moreover, sUA inhibited UCP2 production not only in WT cells, but also in UCP2‐overexpressed H9c2 cells (Figure 3I). When the UCP2 protein levels were up‐regulated, the levels of NLRP3, pro‐caspase 1 and pro‐IL‐1β had no change (Figure 3I). However, UCP2 overexpression could reduce NLRP3, pro‐caspase 1 and pro‐IL‐1β production. Meanwhile, the levels of mIL‐1β and P20 were lower in cells transfected with LV‐UCP2 than those transfected with LV‐NC in the presence of sUA (Figure 3I). Besides, the cell damage induced by sUA was improved by UCP2 overexpression (Figure S5A, B). These data indicated that ROS production induced sUA was mainly dependent on UCP2 levels.

### 3.5 TLR6 knockdown rats improved myocardial damage induced by sUA

**FIGURE 4:**
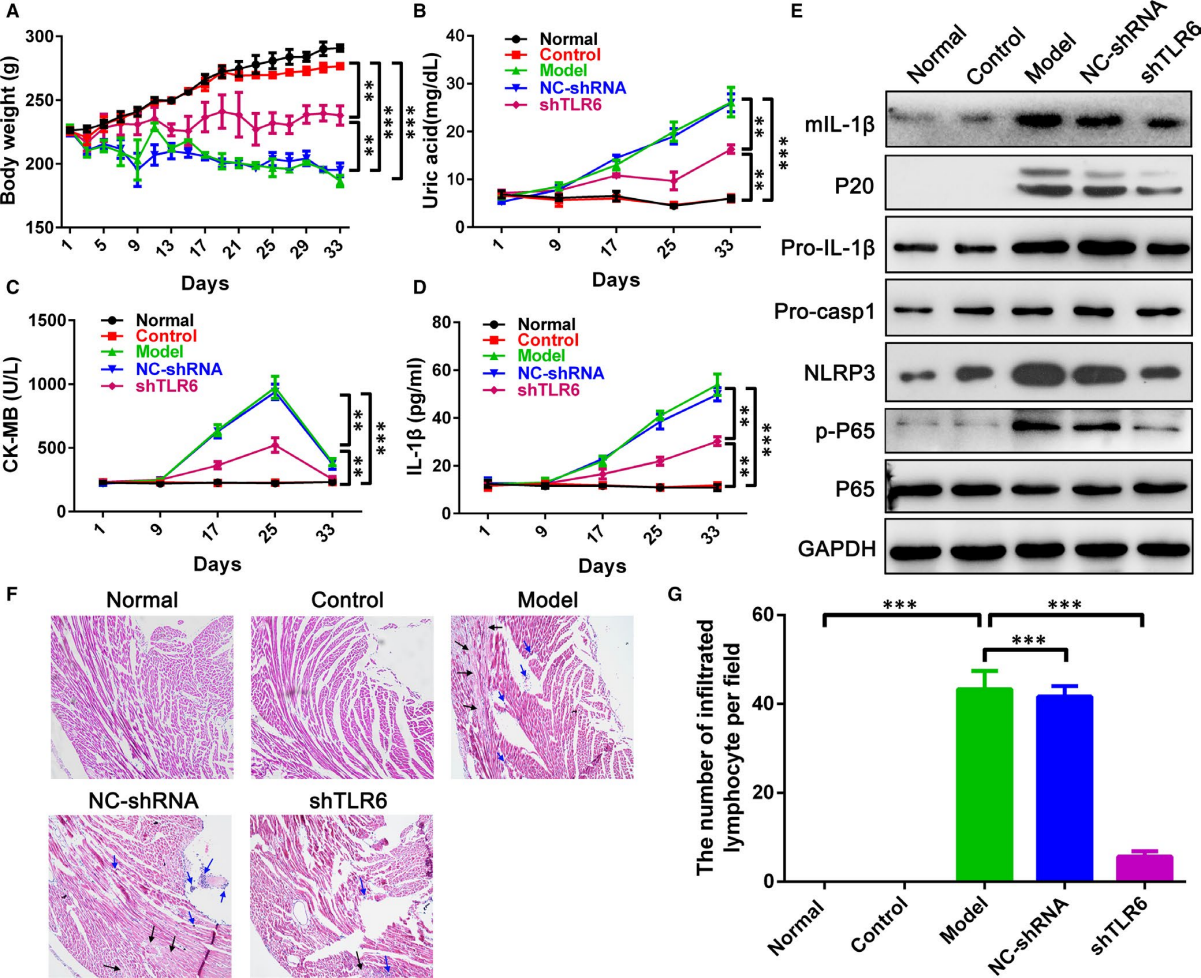
TLR6 knockdown rats improved myocardial damage induced by soluble UA. A, Bodyweight was measured every other day after adenine and ethambutol administration. B‐D, The levels of uric acid (B), CK‐MB (C) and IL‐1β (D) were detected in the serum of rats administrated with adenine and ethambutol. E, The levels of NLRP3 inflammasome components in myocardial tissue were assayed via WB. F, Myocardial tissue sections were stained with H&E fluid to analysed the infiltrating inflammatory cells and myocardial injury. Blue arrows represent infiltrated inflammatory cells and black arrows represent damaged myocardial tissue. G, The quantitative statistical chart of infiltrated inflammatory cells in myocardial tissue. Data are shown as mean ± SEM. **P ≤ 0.01, ***P ≤ 0.001

The preceding experiments had confirmed the role of TLR6 in cardiocyte damage and NLRP3 inflammasome activation induced by sUA. We next examined whether reducing TLR6 level with adenovirus was able to restore myocardial damage and to inhibit NLRP3 inflammasome activation in rats bearing hyperuricemia. Intravenous injection of adenovirus‐shTLR6 12 days later, the levels of *Tlr6* gene were significantly decreased in heart, liver, spleen, lung and kidney tissues (Figure S6A). Meanwhile, the expression of TLR6 protein in heart tissues was also inhibited (Figure S6B). In the model and nc‐shRNA rats, the bodyweight was decreased and the serum UA level was increased after administering with adenine and ethambutol (Figure 4A, B). Compared to model and nc‐shRNA rats, knockdown TLR6 significantly improved the loss of bodyweight and reduced serum UA level (Figure 4A, B). Besides, the CK‐MB level was markedly lower in TLR6 knockdown rats than that in model and nc‐shRNA rats, which was the major marker of cardiocyte damage (Figure 4C). In the meantime, we found that TLR6 knockdown in rats could markedly inhibit pro‐IL‐1β maturation and mIL‐1β secretion in serum and heart tissues (Figure 4D, E). When TLR6 was silenced in rats, the first and second signal pathways of NLRP3 activation were suppressed, including the phosphorylation of P65, NLRP3 level, pro‐IL‐1β level and pro‐caspase 1 maturation (Figure 4D, E).

To further evaluate the role of TLR6 knockdown on cardiocyte damage and NLRP3 inflammasome activation in rats, paraffin sections of heart tissues were stained with haematoxylin and eosin. We found that there were numerous infiltrating lymphocytes and cardiocyte damage in model and NC‐shRNA rats, while TLR6 knockdown obviously inhibited lymphocyte infiltration and improved cardiocyte damage (Figure 4F).

### 3.6 TLR6 knockdown reduced left ventricular remodelling

**FIGURE 5:**
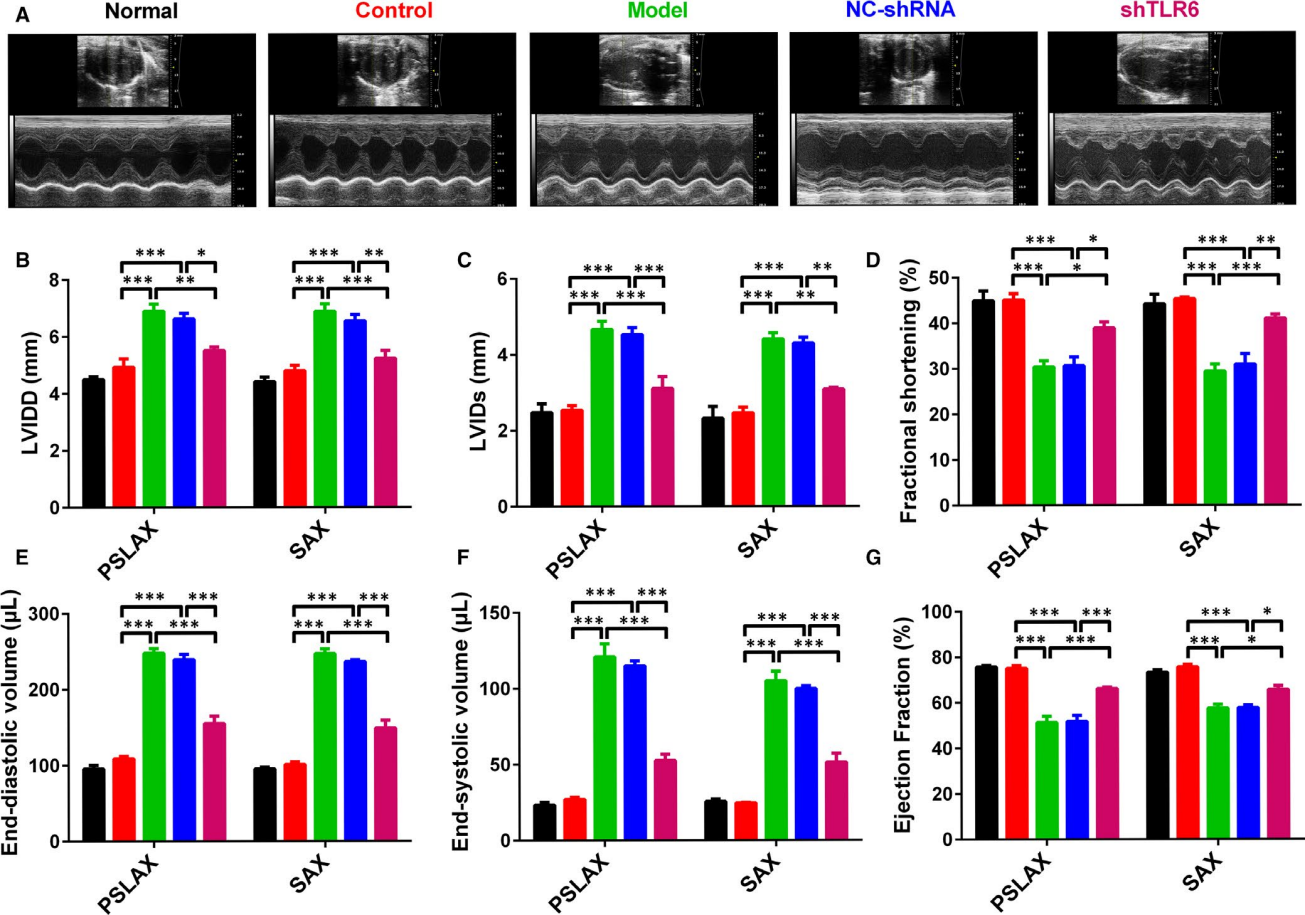
TLR6 knockdown reduced left ventricular remodelling. A, The representative images of the echocardiography which were used to analysed the cardiac function of the rats with hyperuricemia. B‐G, The statistical results of LVIDD, LVIDs, FS, EDV, ESV and EF in the rats with hyperuricemia. Data are shown as mean ± SEM. *P ≤ 0.05, **P ≤ 0.01 and ***P ≤ 0.001

Based on these findings, we have been suggested that TLR6 knockdown contributed to the favourable left ventricular remodelling associated with sUA. To examine this, we analysed the left ventricular remodelling using the echocardiography at 33 days. We found that there were significantly higher LVIDD and LVIDs in model and nc‐shRNA groups through PSLAX and SAX modes, and the fractional shortening in these groups was lower than that in normal and control groups (Figure 5A‐D). Meanwhile, this trend was consistent with end‐diastolic volume (EDV), end‐systolic volume (ESV) and Ejection fraction (EF) (Figure 5E‐G). TLR6 knockouts had smaller EDV, ESV, and higher EF than model and nc‐shRNA groups (Figure 5E‐G). This demonstrated that TLR6 knockouts resulted in an improved heart failure phenotype that ventricular dilation and impaired contractility were partly restored.

## 4 DISCUSSION

**FIGURE 6:**
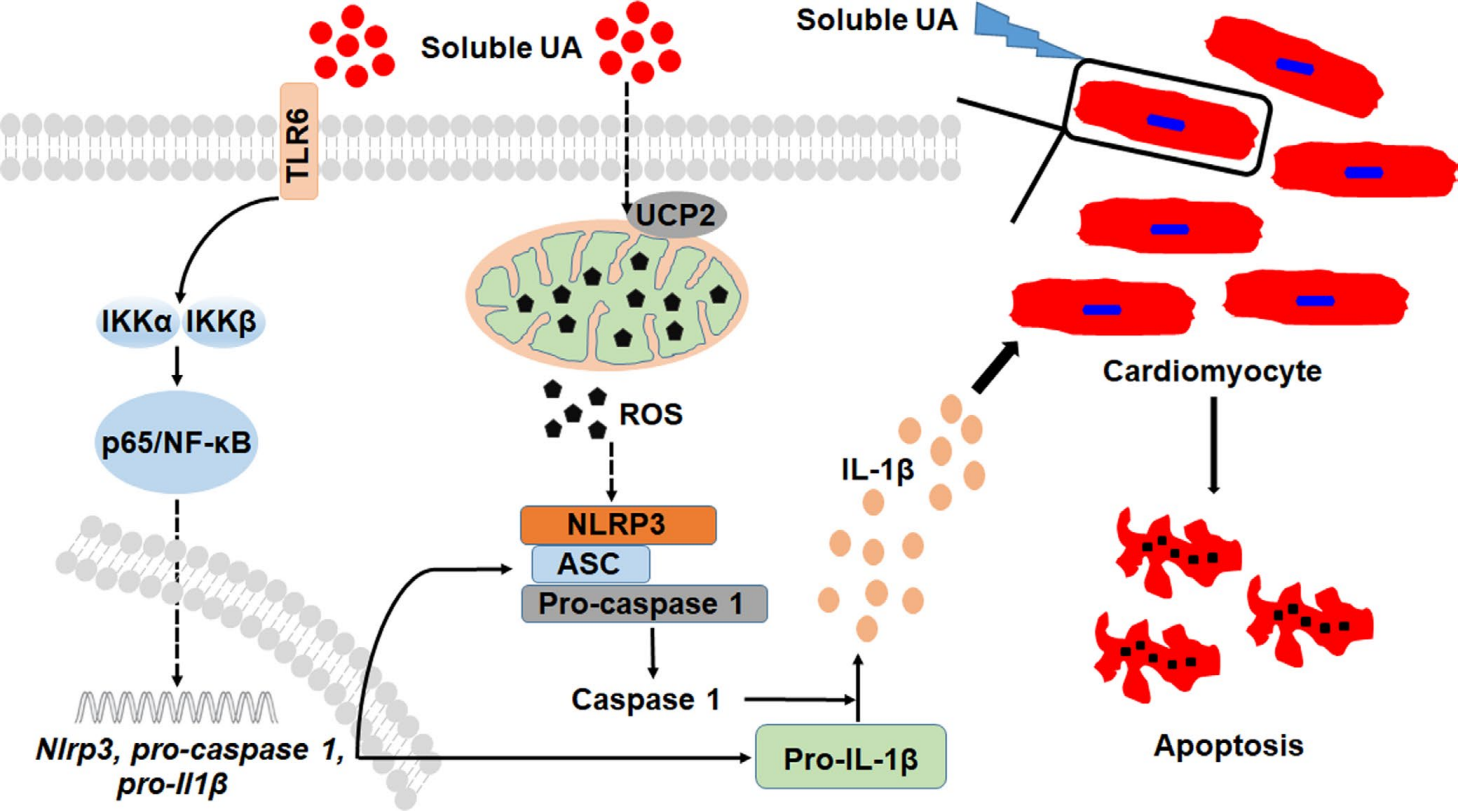
Schematic of identified pathways that soluble UA activates NLRP3 inflammasome in H9c2 cell. Soluble UA up‐regulates TLR6 levels, activates NF‐κB/p65 signal pathway and promotes p65 nuclear transfer, which provides the first signal of NLRP3 inflammasome. Meanwhile, soluble UA down‐regulates the expression of UCP2 that regulates ROS generation, which provides the second signal of NLRP3 inflammasome. After the formation of NLRP3 inflammasome, the protein level of mature IL‐1β is up‐regulated. Excess cytokines (IL‐1β) will damage the cardiomyocytes in paracrine or autocrine form

Crystal formation is a common phenomenon in biological systems, which contributes to the development of skeletons, orientation, navigation and homing.^6^ Moreover, this process is precisely regulated to insure the structure, size and distribution of the crystals. Tiny changes in this process can abnormally activate immune system and damage tissues through NLRP3 inflammasome.^18^, ^28^, ^29^, ^30^ UA crystal, one common kind of crystals and the major feature of hyperuricemia, is strongly related to some diseases, such as gout, chronic renal injury and cardiovascular diseases.^13^, ^18^, ^31^, ^32^ Meanwhile, before crystallization, sUA can also induce the apoptosis of renal proximal tubule epithelial cell and renal fibrosis through activating NLRP3.^33^, ^34^ However, some experiments and clinical researches show that serum UA is a major antioxidant and improves cardiovascular damage.^35^, ^36^ In this study, we aimed to explore whether sUA induced myocardial damage and the mechanism of NLRP3 inflammasome activation in cardiomyocytes induced by sUA. As shown in Figure 6, our work suggested that sUA stimulated the dose‐dependent production of inflammasome‐related molecules, which further induced H9c2 cell apoptosis. Meanwhile, NLRP3 knockdown or inhibiting caspase‐1 activation could reduce the levels of mature IL‐1β and P20, and then improve the cell apoptosis. In addition, NLRP3 inflammasome activation induced by sUA was dependent upon TLR6/NF‐κB/p65 signal pathway and ROS production which was regulated by UCP2. Furthermore, we observed that tlr6 knockdown improved sUA‐induced NRLP3 inflammasome activation, myocardial damage and left ventricular remodelling by inhibiting NLRP3 inflammasome activation in rats.

At present, UA crystal activates NLRP3 inflammasome mainly through frustrated phagocytosis or lysosomal damage.^6^, ^18^, ^20^, ^37^ The lysosomal contents, such as cathepsins and ATP, are released into the cytoplasm and then induce NLRP3 inflammasome activation, although the individual mechanisms remain unknown.^20^, ^38^ Meanwhile, reducing lysosomal damage or inhibiting the release of cathepsins and ATP can inhibit NLRP3 inflammasome activation and improve tissue damage, such as probenecid, which is used to treat reperfusion injury of the brain.^39^ However, when H9c2 cells were stimulated with sUA, the lysosomal was still intact through staining with LysoTracker Green DND‐26 and Alexa Fluor 647 Conjugated dextran. These findings mean that unlike crystals, NLRP3 inflammasome activation induced by sUA is independent of lysosomal damage.

It is well known that crystals can result in the generation of ROS, which induces NLRP3 inflammasome activation.^40^, ^41^, ^42^ Lysosomal damage induced by crystals can cause mitochondrial outer membrane permeabilization (MOMP), which promotes ROS generation and then activates NLRP3 inflammasome.^11^, ^42^ Some antioxidants can partially inhibit NLRP3 activation.^42^ Meanwhile, one recent research shows that sUA can alter mitochondrial components and then induce mitochondrial ROS production.^19^ The mitochondrial damage and oxidative stress are important risk factors for cardiovascular diseases.^43^ ROS can react with NO to form cytotoxic oxidant peroxynitrite and deplete NO, which contributes to cardiovascular diseases.^44^ Meanwhile, ROS is a pivotal link between mitochondrial damage and NLRP3 inflammasome activity.^45^ In H9c2 cells, high glucose could induce the release of cytochrome *c* into the cytosol, which was regulated by ROS.^12^ In addition, through binding to NLRP3, cytochrome *c* activated NLRP3 inflammasome. However, the role and the source of ROS remain controversial. Macrophages from the mice lacking gp91phox did not show the lower level of IL‐1β and responded normally to UA and silica crystals.^30^ In this study, we found that although the lysosomes were intact, sUA could induce mitochondrial damage and ROS production, which was dose‐dependent. Meanwhile, the ROS scavenger, NAC could reduce the level of ROS, inhibit NLRP3 inflammasome activation and then prevent pro‐IL‐1β maturation. Interestingly, although NAC could normalize the level of ROS, NLRP3 inflammasome activity was not completely inhibited. Indeed, mitochondria have many mechanisms that activate NLRP3 inflammasome, such as mitochondrial DNA, AMP/ATP ratios, mitochondrial Ca^2+^ overload, the location of mitochondrial protein, mitochondrial dynamics and transport.^42^, ^45^

When ROS is generated in the cells, it can directly promote the formation of the caspase‐1/ASC complex and then activate NLRP3 inflammasome. Meanwhile, through dissociating TXNIP from TXNIP/TXN complex or inducing the release of cytochrome *c*, ROS can indirectly activate NLRP3 inflammasome.^12^, ^46^, ^47^ These studies have partly described the downstream effects of sUA, while the upstream mechanisms regulating ROS production are still uncertain in the cardiomyocytes. Indeed, sUA induces the generation of HMGB‐1, which can bind to RAGE and induce an oxidative status. Through blocking the HMGB‐1/RAGE signalling pathway, soluble RAGE can reduce the level of ROS and improve diabetic mice.^48^, ^49^ Furthermore, some mitochondrial proteins, such as TSPO, UCP2, Slc25a1 and VDAC, can regulate mitochondrial ROS production.^50^, ^51^, ^52^, ^53^ Inhibiting these proteins can regulate NLRP3 inflammasome activation. We observed that sUA could inhibit the generation of UCP2, while other related proteins had no obvious change. When the level of UCP2 was elevated by LV‐UCP2, ROS production was dramatically reduced and NLRP3 inflammasome activation was significantly inhibited. Although some researches have shown that the level of UCP2 is elevated when NLRP3 inflammasome is activated, the results are not credible in our opinion. In these researches, the authors identify that UCP2 can prevent ROS production. However, when the cells are treated with LPS and ATP, the levels of UCP2 and ROS are simultaneously elevated.^53^, ^54^

The NLRP3 inflammasome activation requires two signals, and the first signal is capable to elevate the levels of *NLRP3*, *IL‐1β*, *caspase‐1* genes and proteins, which is mainly dependent on TLR. Besides that cholesterol crystal needs TLR4 to provide the first signal, other crystals can directly engage cellular membranes independent of any known cellular receptor.^6^, ^55^ Recently, some researches have shown that UA crystal can be recognized by the complement system and induce the generation of C5a, which provides the first signal of NLRP3 inflammasome activation.^56^, ^57^ Meanwhile, the cells can also recognize UA crystal as DAMP through TLR2 or TLR4 and then promote the maturation of pro‐IL‐1β.^6^ Moreover, TLR4 has also been identified as a receptor for sUA and induces inflammatory reaction.^58^ sUA can elevate the expression of TLR4 in human primary renal proximal tubule epithelial cells and PBMCs.^59^, ^60^ The TLR4 inhibitor or silencing the adaptor molecule myeloid differentiation factor 88 (MyD88) can dramatically block NLRP3 inflammasome activation, reduce the level of mature IL‐1β.^19^, ^27^ In H9c2 cells, we found that sUA could elevate the levels of TLR4 and TLR6. Because the role of TLR4 had been expounded in many researches, TLR6 attracted our attention. When the level of TLR6 was knockdown with siRNA, the protein levels of NLRP3, pro‐IL‐1β and pro‐caspase 1 were significantly reduced. Meanwhile, TLR6 knockdown cells displayed a decrease in the levels of mIL‐1β and P20. The downstream signal of TLR6 induced by sUA was mainly related to NF‐κB/p65 and independent of TAK1/MKK4/JNK and MKK6/p38 MAPK signals. In vivo, tlr6 knockdown improved myocardial damage and left ventricular remodelling by inhibiting NLRP3 inflammasome activation in rats.

Altogether, our data demonstrated that sUA could induce cardiomyocyte damage though activating NLRP3 inflammasome in a TLR6/NF‐κB/p65‐dependent manner. Meanwhile, mitochondrial ROS production regulated by UCP2 provided the second signal of NLRP3 inflammasome. The observation that NLRP3 inflammasome activation could be induced by sUA might provide a therapeutic strategy for myocardial damage induced by UA.

## CONFLICT OF INTEREST

The authors declare no conflict of interests.

## AUTHOR CONTRIBUTION

**Hailong Zhang:** Conceptualization (supporting); Data curation (equal); Formal analysis (equal); Funding acquisition (supporting); Investigation (lead); Methodology (equal); Project administration (equal); Software (supporting); Validation (equal); Writing‐original draft (lead); Writing‐review & editing (equal). **Yuting Ma:** Data curation (equal); Formal analysis (equal); Investigation (supporting); Methodology (equal); Project administration (equal); Software (lead); Validation (equal); Writing‐review & editing (supporting). **Run Cao:** Data curation (equal); Formal analysis (equal); Investigation (supporting); Methodology (equal); Project administration (equal); Software (supporting); Validation (equal); Writing‐review & editing (supporting). **Guanli Wang:** Data curation (supporting); Methodology (supporting); Project administration (supporting); Software (supporting); Validation (supporting). **Shaowei Li:** Data curation (supporting); Methodology (supporting); Project administration (supporting). **Yue Cao:** Data curation (supporting); Methodology (supporting); Project administration (supporting). **Hao Zhang:** Data curation (supporting); Formal analysis (supporting); Software (supporting). **Meichen Liu:** Project administration (supporting); Software (supporting). **Guangchao Liu:** Formal analysis (supporting). **Jun Zhang:** Formal analysis (supporting). **Shulian Li:** Formal analysis (supporting). **Yaohui Wang:** Conceptualization (lead); Resources (equal); Supervision (equal); Writing‐review & editing (equal). **Yuanfang Ma:** Conceptualization (supporting); Funding acquisition (lead); Resources (lead); Supervision (equal); Writing‐review & editing (equal).

## Supporting information

Supplementary Material

## Data Availability

The data that support the findings of this study are available from the corresponding author upon reasonable request.
