# Supplementary material for: Soluble uric acid induces myocardial damage through activating the NLRP3 inflammasome
Source: J Cell Mol Med. 2020 Jun 18;24(15):8849–61. doi: 10.1111/jcmm.15523 (PMC7412683; doi:10.1111/jcmm.15523)
Supplement: Supplementary file 1 — Supplementary Material [file JCMM-24-8849-s001.doc]

**Soluble uric acid induces myocardial damage through activating the NLRP3 inflammasome**

Hailong Zhang^1*^, Yuting Ma^1*^, Run Cao^1*^, Guanli Wang^2^, Shaowei Li^1^, Yue Cao^1^, Hao Zhang^1^, Meichen Liu^1^, Guangchao Liu^1^, Jun Zhang^1^, Shulian Li^1^, Yaohui Wang^1#^, Yuanfang Ma^1#^

**1. Supplementary methods**

**1.1. Animal experiments**

First, twenty rats were randomly divided into two groups, one group was intravenously administrated with Adenovirus-null (Ad-null, MOI=100), another group was intravenously administrated with Adenovirus-shTLR6 (Ad-shTLR6). Twelve days later, the level of TLR6 in the tissues was detected by RT-qPCR and on western blot.

Second, the rats administrated with adenovirus were intragastrically administrated with 100 mg/kg of adenine (2.5ml/kg) and 250 mg/kg of ethambutol (2.5ml/kg) every day. The adenine was dissolved in PBS, and the ethambutol was dissolved in 0.5% sodium carboxymethyl cellulose. Meanwhile, Normal group, Model group, and Solvent control (SC) group were established. Model group and SC group were intragastrically administrated with 100 mg/kg of adenine and 250 mg/kg of ethambutol every day, and SC group was also intragastrically administrated with 2.5ml/kg 0.5% sodium carboxymethyl cellulose and PBS. Once a week, the level of serum UA and body weight were recorded.

Third, color Doppler ultrasound was used to detect the cardiac function of rats at 33 days. Then, the rats were sacrificed under anaesthesia. Blood was obtained from the carotid artery to select the serum. After irrigation, the heart tissue was rapidly frozen with liquid nitrogen to extract the related genes and proteins. Meanwhile, the heart tissue was embedded in 4% paraformaldehyde for pathological analysis.

**2. Supplementary Figures**

**
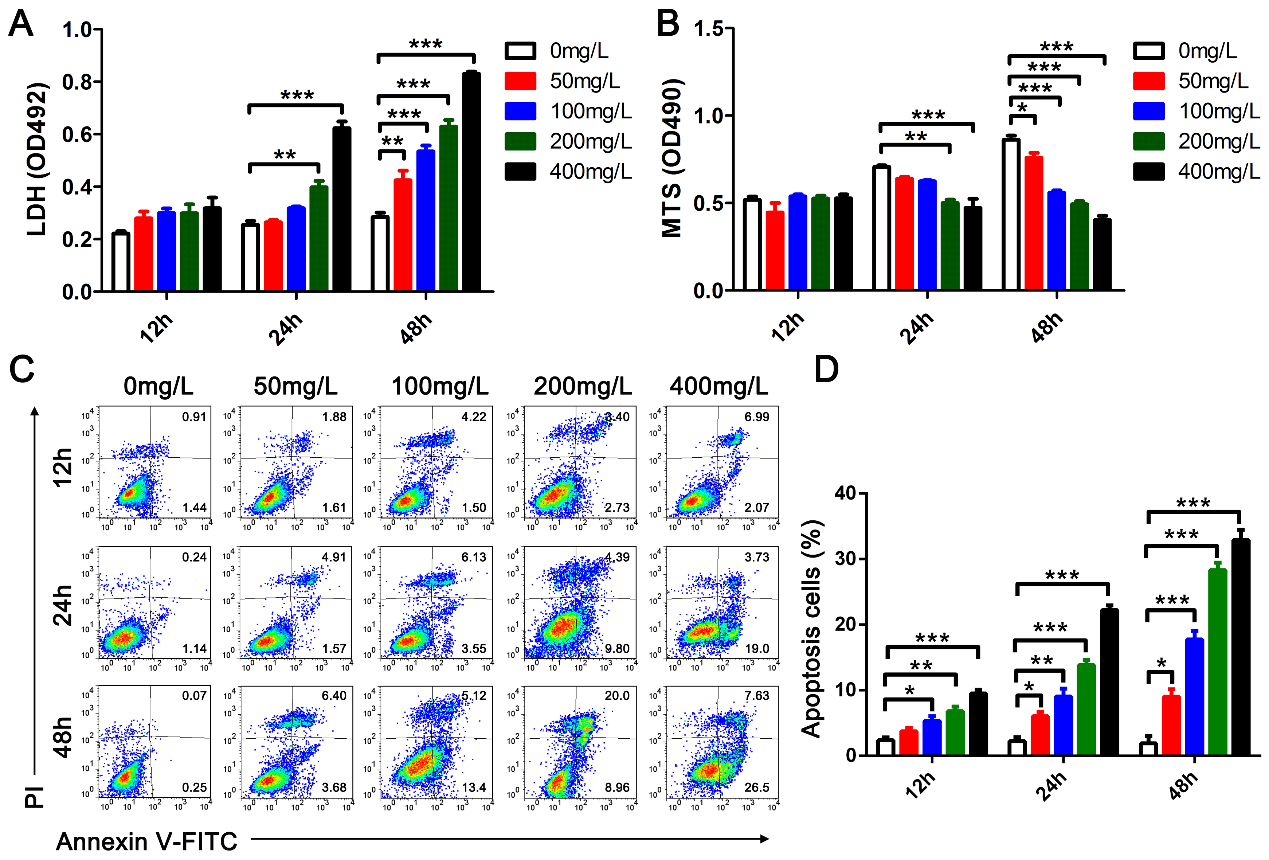
**

**Sup Figure 1.** Soluble UA induced H9c2 cell damage and apoptosis. **A-B** LDH (A) and MTS (B) were used to detect H9c2 cell damage which was treated with soluble UA for serial hours (12, 24 and 48 h). **C-D** The representative images of FCM of H9c2 cells stimulated with soluble UA for serial hours (C) and the statistical result of apoptotic rate (D). Data are shown as mean ± SEM. **P* ≤ 0.05, ***P* ≤ 0.01, ****P* ≤ 0.001


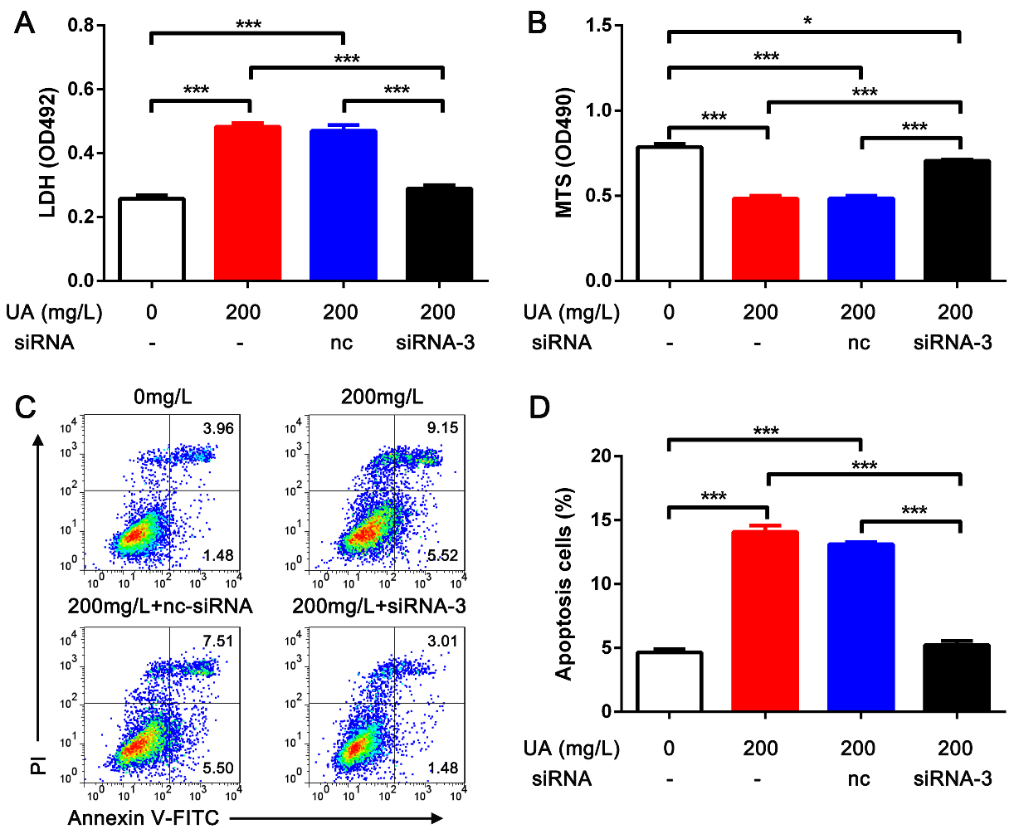


**Sup Figure 2.** Silencing TLR6 improved H9c2 cell damage and apoptosis induced by soluble UA. **A-B** LDH (A) and MTS (B) were used to detect H9c2 cell damage induced by soluble UA after TLR6 was silenced. **C-D** The representative images of FCM of H9c2 cells stimulated with soluble UA after TLR6 was silenced (C) and the statistical result of apoptotic rate (D). Data are shown as mean ± SEM. **P* ≤ 0.05, ****P* ≤ 0.001.


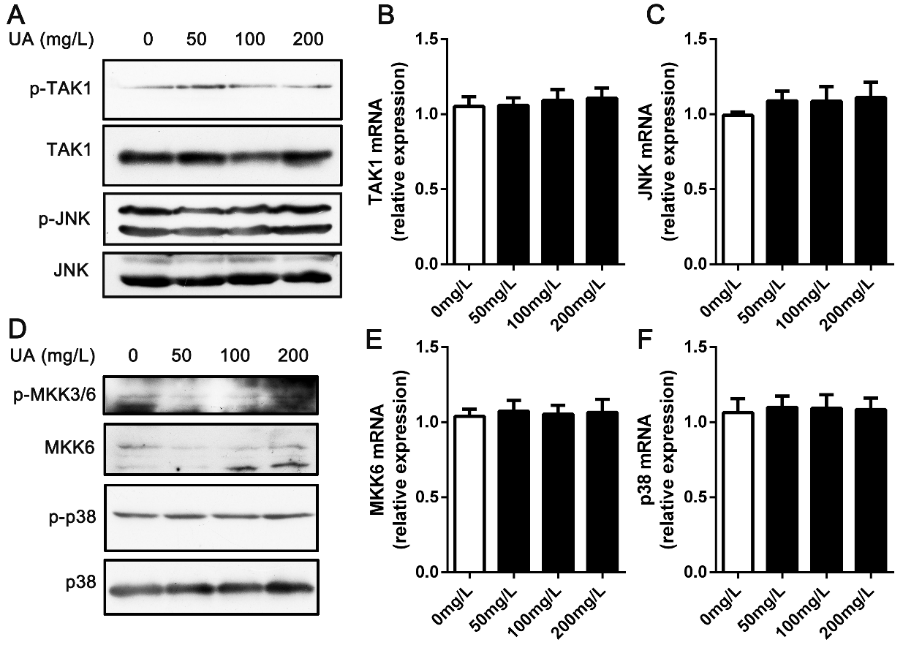


**Sup Figure 3**. Soluble UA did not affect the activation of TAK1/MKK4/JNK and MKK6/p38 MAPK signal pathways. **A** The protein levels of p-TAK1, TAK1, p-JNK and JNK were detected by WB in H9c2 cells treated with soluble UA. **B-C** The gene levels of TAK1 (**B**) and JNK (**C**) were analyzed by RT-qPCR in H9c2 cells treated with soluble UA. **D** The protein levels of p-MKK3/6, MKK6, p-p38 and p38 were analyzed by WB in H9c2 cells treated with soluble UA. **E-F** The gene levels of MKK6 (**E**) and p38 (**F**) were analyzed by RT-qPCR in H9c2 cells treated with soluble UA. Data are shown as mean ± SEM.


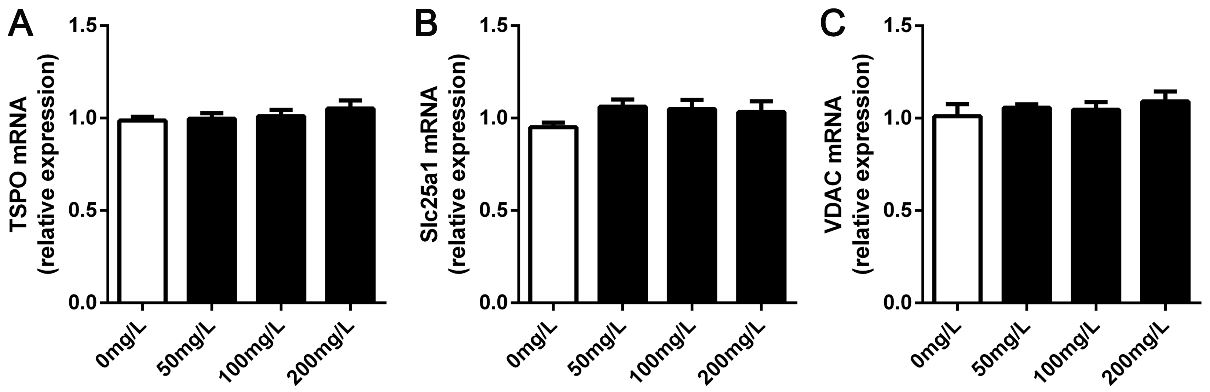


**Sup Figure 4**. The levels of mitochondrial membrane component-related genes in H9c2 cells. **A-C** The gene levels of *Tspo* (**A**), *Slc25a1* (**B**) and *VDAC* (**C**) in H9c2 cells treated with soluble UA.


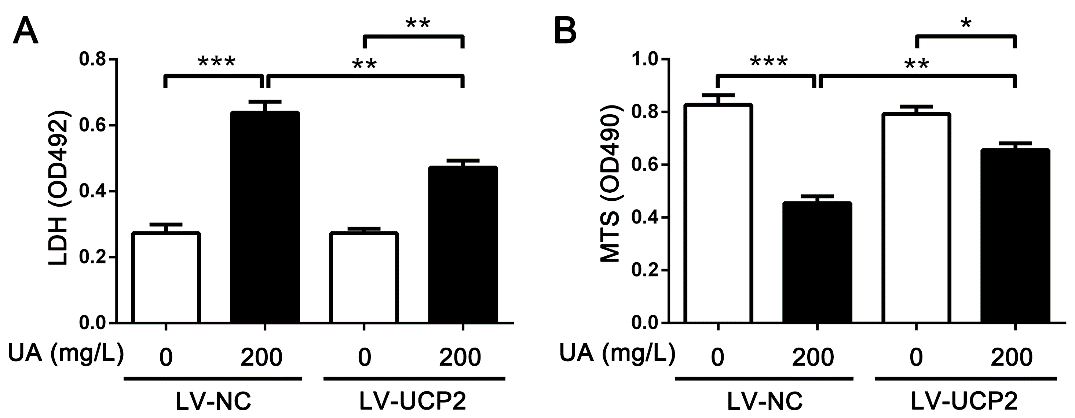


**Sup Figure 5**. Overexpressing UCP2 improved cell damage induced by soluble UA. **A-B** The cell damage induced by soluble UA was analyzed by LDH (**A**) and MTS (**B**) in H9c2 cells transfected with LV-UCP2. Data are shown as mean ± SEM. **P* ≤ 0.05, ***P* ≤ 0.01, ****P* ≤ 0.001.


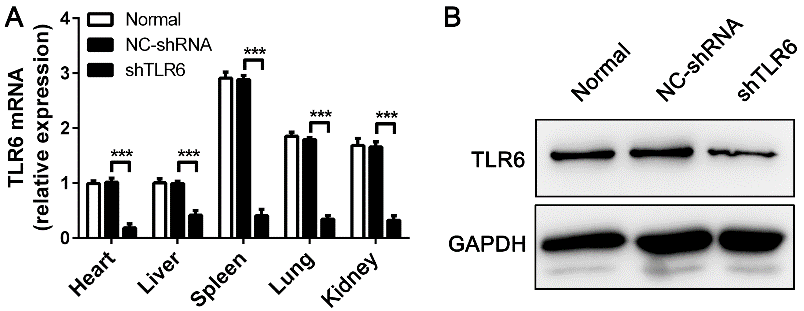


**Sup Figure 6.** The gene and protein levels of TLR6 in the rats. **A** The gene level of *tlr6* in the hearts, livers, spleens, lungs and kidneys of the rats administrated with adenovirus-shTLR6 for twelve days. **B** The protein level of TLR6 in the heart of the rats administrated with adenovirus-shTLR6 for twelve days.

**3. Supplementary Tables**

**Supplementary Table 1.** Sequences of primers for real-time RT-PCR.

| Gene name | Forward | Reverse |
| --- | --- | --- |
| *gapdh* | ACGGGAAACCCATCACCAT | CTCGTGGTTCACACCCATCA |
| *nlrp3* | AGCTGCTCTTTGAGCCTGAG | TCTGCTAGGCTCTTTGGTGC |
| *asc* | TTGCTGGATGCTCTGTATG | CCAAGTAGGGCTGTGTTTGC |
| *Caspase-1* | GACCGAGTGGTTCCCTCAAG | GACGTGTACGAGTGGGTGTT |
| IL-1β | GGCCTCAAGGGGAAGAATC | ATGTCCCGACCATTGCTGTT |
| *Tlr1* | AGGTACAGTGATGCGGGAAG | TGACCTTGGGAGGTAAGCAC |
| *Tlr2* | GAGTCTGCTGTGCCCTTCTC | GCTTTCTTGGGCTTCCTCTT |
| *Tlr3* | CCCCCTTCCAACTCCTATTC | TCGATGCACTGAAACATTCC |
| *Tlr4* | TGGCATCATCTTCATTGTCC | CAGAGCATTGTCCTCCCACT |
| *Tlr5* | ACATCAAAGATCCCGACCAG | TTCAGCGTCTCAAACAGTCG |
| *Tlr6* | GTCTCCCCACTTCATCCAGA | ATGGGTTCCAGCAAGATCAG |
| *Tlr7* | GGAGCTGGTGGTTAAATTGG | GCTGTATGCTCTGGGAAAGG |
| *Tlr8* | TCTATTTGGGCTGGAACTGC | GAGGCACGGAGAAAAGGTTA |
| *Tlr9* | CCTTATGATGCCTTCGTGGT | ATCTCGGTCCTCCAGACACA |
| *Tspo* | GGTGGACCTCATGCTTGTCA | CCTCGCCGACCAGAGTTATC |
| *Slc25a1* | CAGAGGCAGTGGTAGTCGTG | GTGAGGCCTTGGTATGTCCC |
| *VDAC* | TGGAACACAGACAACACCCT | AACCCTCATAGCCAAGCACC |
